# An innovative subdivision collocation algorithm for heat conduction equation with non-uniform thermal diffusivity

**DOI:** 10.1038/s41598-024-73772-3

**Published:** 2024-10-08

**Authors:** Syeda Tehmina Ejaz, Safia Malik, Jihad Younis, Rahma Sellami, Kholood Alnefaie

**Affiliations:** 1 Department of Mathematics, The Government Sadiq College Women University, Bahawalpur, 63100 Pakistan; 2 Department of Mathematics, Aden University, P.O.Box 6014, Aden, Yemen; 3 Department of Computer Science, Applied College, Northern Border University, Rafha, 91911 Saudi Arabia; 4 Department of Mathematics, College of Science, Taibah University, Madinah, 42353 Saudi Arabia

**Keywords:** Partial differential equation, Heat conduction equation, Subdivision scheme, Collocation method, Stability, Error, Engineering, Mathematics and computing, Physics

## Abstract

This paper presents a subdivision collocation algorithm for numerically solving the heat conduction equation with non-uniform thermal diffusivity, considering both initial and boundary conditions. The algorithm involves transforming the differential form of the heat conduction equation into a system of equations and discretizing the time variable using the finite difference formula. The numerical solution of the system of heat conduction equations is then obtained. The feasibility of the algorithm is verified through theoretical and numerical analyses. Additionally, numerical and graphical representations of the obtained numerical solutions are provided, along with a comparison to existing methods. The results demonstrate that our proposed method outperforms the existing methods in terms of accuracy.

## Introduction

This paper introduces the applications of the subdivision collocation algorithm for solving the following heat conduction problem:

1 $$\begin{aligned} \frac{\partial u}{\partial t}=\alpha (x,t)\frac{\partial ^{2} u}{\partial x^2}+\xi (x, t), \qquad 0\le x \le \mathbb {L} \quad \& \quad {t >0}, \end{aligned}$$

with initial and boundary conditions

2 $$\begin{aligned} & u(x,0)=h(x) \end{aligned}$$

3 $$\begin{aligned} & u(0,t)=g_{1}(t), u(\mathbb {L},t)=g_{2}(t) \end{aligned}$$

where *u*(*x*, *t*) is the temperature distribution along the length $\mathbb {L}$ over time *t*, $\xi (x, t)$ is the heat source, $\alpha (x,t)$ is the thermal diffusivity varying with the space and temporal coordinates. This equation in the interval $[0, \mathbb {L}]$ satisfies the boundary conditions $g_{1}(t)$ and $g_{2}(t)$ which are known functions. There are different types of materials in which thermal diffusivity $\alpha (x,t)$ varies with time and both space and time coordinates. When thermal diffusivity depends on time, it can occur in materials such as concrete and aging metals. If thermal diffusivity depends on both space and time, examples include composite materials and functionally graded materials.

Heat conduction plays a crucial role in engineering, particularly in areas where thermal management and heat transfer are essential considerations. It finds specific applications in various engineering fields such as electronics and electrical engineering, energy systems and power plants, aerospace and aviation, automotive engineering, HVAC systems and building engineering, industrial processes and manufacturing, and thermal fluid systems. The heat equation with variable coefficients provide a fundamental framework for analyzing and predicting heat transfer phenomenon in system where the thermal properties varies spatially or temporally or both at the same time. Many researchers made great contributions to deal the heat equation with uniform and non uniform thermal diffusivity analytically and numerically. In the past; numerical method based on rational approximation to the matrix exponential functions was employed to solve the heat equation with variable coefficients^1^. Parabolic partial differential equations with variable coefficients was solved analytically in^2^. These given methods were employed to solve the heat conduction equation; generalized forward difference method for transient heat conduction^3^, meshless method for solving the backward heat conduction problem^4^, Cubic B spline collocation method^5–7^ and a new iteration method which identified the time dependant coefficient in heat conduction^8^. Similarly a multiscale algorithm was proposed to solve the heat conduction equation^9^. Variational iteration method was introduced to handle the heat transfer equation in^10^. Meshless BEM was presented in^11^, to deal with isotropic heat conduction problems in which heat was generated and conductivity varies along space coordinates. Finite element approximation was introduced using web splines to solve the heat equation in^12^. In a series of papers different methods was used for heat conduction problem such as multiscale finite element method for a free boundary problem^13^, radial integration BEM^14^, spectral method^15^, hat wavelet transform^16^ and for unsteady heat conduction equation a high order scheme was given in^17^.

The aim of this paper is to find a solution for the heat conduction equation using subdivision collocation method. In recent literature, stationary binary subdivision schemes using radial basis function interpolation was introduced by^18^. Furthermore, the subdivision collocation algorithm has been successfully applied to find solutions for various ordinary differential equations^19–22^, and recently, its application to solving partial differential and fractional equations has been explored^23–26^. These algorithms provide rapid convergence, high accuracy, and stability compared to existing methods. Subdivision-based collocation algorithms are particularly noteworthy for their rapid convergence and high accuracy. This motivates the application of the subdivision collocation algorithm to the heat conduction equation with variable coefficients to obtain its numerical solutions.

This paper is structured as follows. In “Subdivision scheme” section presents the essential characteristics of the 6-point binary subdivision system. In “Subdivision collocation algorithm for heat conduction equation” section introduces the subdivision collocation algorithm, which is constructed based on the concept of subdivision schemes. In “Stability analysis and errors evaluation” section includes the stability and errors estimation of the proposed method. In “Comparison of numerical example with existing methods” section demonstrate the effectiveness and accuracy of the proposed method through numerical experiments, along with comparison of the solutions obtained by different methods. Finally, the last section concludes the paper with a brief summary.

## Subdivision scheme

If the successive control points of the polygon at $k^{th}$ and $(k+1)^{th}$ level are $P^{k}_i$ and $P^{k+1}_i$, then the six points interpolating binary subdivision scheme proposed by^18^ is defined as

4 $$\begin{aligned} \left\{ \begin{array}{l} P_{2i}^{k+1}=P_{i}^k \\ P_{2i+1}^{k+1}=\omega (P_{i-2}^{k}+P_{i+3}^{k})+(-\frac{1}{16}-3\omega )(P_{i-1}^{k}+P_{i+2}^{k})\\ \qquad \quad +(\frac{9}{16}+2\omega )(P_{i}^{k}+P_{i+1}^{k}). \end{array}\right. \end{aligned}$$

This scheme produces $C^{2}-$ continuous curve for $\omega =\frac{3}{256}$. The scheme reproduces polynomial curves of degree seven, approximation order is four, cardinal support of this scheme is $[-4,4]$. The fundamental solution and two-scale relationship of (4) are defined in (5) and (6) accordingly.

5 $$\begin{aligned} \mathscr {D}(i)=\left\{ \begin{array}{l} 0, \quad \text{ if }\quad i\ne 0,\\ 1, \quad \text{ if }\quad i=0 \end{array}\right. \end{aligned}$$

and

6 $$\begin{aligned} \mathscr {D}(x)=\Sigma a_k \mathscr {D}(2x-k), \end{aligned}$$

The first two derivatives of (4) at integral support are given in (7).

7 $$\begin{aligned} \left\{ \begin{array}{l} \mathscr {D}^{'}(0)=0,\qquad \qquad \qquad \qquad \mathscr {D}^{''}(0)=-\frac{295}{28},\\ \mathscr {D}^{'}(\pm 1)=\mp \frac{272}{365},\qquad \qquad \quad \,\ \mathscr {D}^{''}(\pm 1)=\frac{712}{105}, \\ \mathscr {D}^{'}(\pm 2)=\mp \frac{53}{365},\qquad \& \qquad\,\  \mathscr {D}^{''}(\pm 2)=-\frac{184}{105},\\ \mathscr {D}^{'}(\pm 3)=\mp \frac{16}{1095},\quad \qquad \qquad \mathscr {D}^{''}(\pm 3)=\frac{8}{35},\\ \mathscr {D}^{'}(\pm 4)=\mp \frac{1}{2920},\quad \qquad\qquad \mathscr {D}^{''}(\pm 4)=\frac{3}{280}. \end{array}\right. \end{aligned}$$

## Subdivision collocation algorithm for heat conduction equation

In this section, we have constructed a subdivision collocation method for solving the heat conduction equation. This method is based on the derivatives of the subdivision scheme (4) and its fundamental solution.

### Formulation of algorithm

Let the approximate solution of (1), with unknown $\{v_{j}\}$ is given below

8 $$\begin{aligned} & u(x,t)= \sum ^{{i+4}}_{{j=i-4}} a^{H}_{m-j}v_{j}\mathscr {S}_{2, x_j},\qquad 0\le x\le \mathbb {L}, \end{aligned}$$

9 $$\begin{aligned} & \left\{ \begin{array}{l} {a^{H}_{m-j}=1}, \quad \text{ if }\quad m\ne j,\\ {a^{H}_{m-j}>1}, \quad \text{ if }\quad m=j \end{array}\right. \end{aligned}$$

where $a^{H}_{m-j}$ is positive integer, $\mathscr {N}$ must be greater or equal to 4, $h=\frac{\mathbb {L}}{\mathscr {N}}, i=m=0,1,2,\cdots , \mathscr {N}$, $\mathscr {S}_{2,x_j}=\mathscr {D}(\frac{x_{i}-x_{j}}{h})$ is the basis function of $C^2-$ continuous scheme, $v_{j}'s$ are the time dependent unknowns to be determined. The approximate solution (8) at the point $(x_i,t_k)$ over the interval $[x_i,x_{i+1}]$ denoted by $u^{k}_{i}$ and defined as

10 $$\begin{aligned} u^{k}_{i}=\sum \limits ^{i+4}_{{j=i-4}}a^{H}_{m-j}v^{k}_{j}{\mathscr {S}_{2,x_j}}, \qquad i=0,1,2, \cdots ,\mathscr {N}. \end{aligned}$$

From (10), we obtain

11 $$\begin{aligned} D^{2}u^{k}_{i}=\frac{1}{h^2}\sum \limits ^{i+4}_{{j=i-4}}a^{H}_{m-j}v^{k}_{j}\mathscr {S}''_{2,x_j}, \qquad i=0,1,2, \cdots ,\mathscr {N}. \end{aligned}$$

To obtain the approximations of the solutions, the values of ${\mathscr {S}}_{2,x_j}$ and ${\mathscr {S}}''_{2,x_j}$ at the knots are needed. Since the values vanish at all other knots, they are omitted from (5) and (7). The approximations of the solutions of (1.1) at $t_{j+1}th$ time level can be considered as by^7^

12 $$\begin{aligned} (u_{t})^{k}_{i}+\alpha (x,t)(1-\theta )f^{k}_{i}+\alpha (x,t)\theta f_{i}^{k+1}-\xi ^k_{i}(x_i,t)=0, \end{aligned}$$

where

13 $$\begin{aligned} f^{k}_{i}=-(u_{xx})^{k}_{i}, \end{aligned}$$

superscripts *k* and $k+1$ are successive time levels $k=0,1,2, \cdots$ now discretize the time derivative by a first order accurate forward difference scheme and rearranging the equations we obtain

14 $$\begin{aligned} (u_{t})^{k}_{i}=\frac{1}{ \Delta t}\left[ u^{k+1}_{i}-u^{k}_{i}\right] , \end{aligned}$$

where $\Delta t$ is the time step. Using (10), (11), (13) and (14) in (12) we get

$$\begin{aligned} \frac{1}{ \Delta t}\left[ u^{k+1}_{i}-u^{k}_{i}\right] - \frac{\alpha (x,t)(1-\theta )}{ h^2}\sum \limits ^{{i+4}}_{{j=i-4}}v^{k}_{j}a^{H}_{m-j}\mathscr {S}^{''}(i-j)- \frac{\alpha (x,t)(\theta )}{ h^2}\times \sum \limits ^{{i+4}}_{{j=i-4}}v^{k+1}_{j}a^{H}_{m-j}\mathscr {S}^{''}(i-j)-\xi ^k_{i}(x_i,t)=0, \end{aligned}$$

this implies

15 $$\begin{aligned} u^{k+1}_{i}- \Delta {t} \frac{\alpha (x,t)(\theta )}{h^2}\sum \limits ^{{i+4}}_{{j=i-4}}v^{k+1}_{j}a^{H}_{m-j}\mathscr {S}^{''}(i-j)=u^{k}_{i}+ \Delta {t} \frac{\alpha (x,t)(1-\theta )}{h^2}\times \sum \limits ^{{i+4}}_{{j=i-4}}v^{k}_{j}a^{H}_{m-j}\mathscr {S}^{''}(i-j)+ \Delta t \xi ^k_{i}(x_i,t). \end{aligned}$$

Note that the system becomes an explicit scheme when $\theta =0$, a fully implicit scheme when $\theta =1$, and a mixed scheme of Crank-Nicolson when $\theta =0.5$^7^. Here, Crank-Nicolson approach is used. Hence, (15) takes the form

16 $$\begin{aligned} u^{k+1}_{i}-\frac{\alpha (x,t) \Delta {t}}{2h^2}\sum \limits ^{{i+4}}_{{j=i-4}}v^{k+1}_{j}a^{H}_{m-j}\mathscr {S}^{''}(i-j)=u^{k}_{i}+\frac{\alpha (x,t) \Delta {t}}{2h^2} \sum \limits ^{{i+4}}_{{j=i-4}}v^{k}_{j}a^{H}_{m-j}\mathscr {S}^{''}(i-j)+ \Delta t \xi ^k_{i}(x_i,t), \end{aligned}$$

by substituting $i=m=0$, into the system of equations (16), we get

17 $$\begin{aligned} u^{k+1}_{0}-\frac{\alpha (x,t) \Delta {t}}{2h^2}\sum \limits ^{4}_{j=-4}v^{k+1}_{j}a^{H}_{-j}\mathscr {D}^{''}(-j)=u^{k}_{0}+\frac{\alpha (x,t) \Delta {t}}{2h^2}\sum \limits ^{4}_{j=-4}v^{k}_{j}a^{H}_{-j}\mathscr {D}^{''}(-j)+ \Delta t \xi ^k_{0}(x_0,t). \end{aligned}$$

Now substituting $i=m=1$, into the system of equations (16), we get

18 $$\begin{aligned} u^{k+1}_{1}-\frac{\alpha (x,t) \Delta {t}}{2h^2}\sum \limits ^{5}_{j=-3}v^{k+1}_{j}a^{H}_{1-j}\mathscr {D}^{''}(1-j)=u^{k}_{1}+\frac{\alpha (x,t) \Delta {t}}{2h^2}\times \sum \limits ^{5}_{{j=-3}}v^{k}_{j}a^{H}_{1-j}\mathscr {D}^{''}(1-j)+ \Delta t \xi ^k_{1}(x_1,t). \end{aligned}$$

Similarly for $i=m=\mathscr {N}-1$, the system of equations (16) becomes

19 $$\begin{aligned} & u^{k+1}_{\mathscr {N}-1}-\frac{\alpha (x,t) \Delta {t}}{2h^2}\sum \limits ^{\mathscr {N}+3}_{j=N-5}v^{k+1}_{j}a^{H}_{\mathscr {N}-1-j}\mathscr {D}^{''}(N-1-j)= u^{k}_{\mathscr {N}-1}+\frac{\alpha (x,t) \Delta {t}}{2h^2}\times \nonumber \\ & \sum \limits ^{\mathscr {N}+3}_{j=\mathscr {N}-5}v^{k}_{j} a^{H}_{\mathscr {N}-1-j}\mathscr {D}^{''}(\mathscr {N}-1-j)+ \Delta t \xi ^k_{\mathscr {N}-1}(x_{\mathscr {N}-1},t). \end{aligned}$$

Similarly for $i=m=\mathscr {N}$, the system of equations (16) becomes

20 $$\begin{aligned} & u^{k+1}_{\mathscr {N}}-\frac{\alpha (x,t) \Delta {t}}{2h^2}\sum \limits ^{{\mathscr {N}+4}}_{{j=\mathscr {N}-4}}v^{k+1}_{j}a^{H}_{\mathscr {N}-j}\mathscr {D}^{''}(\mathscr {N}-j) =u^{k}_{\mathscr {N}}+\frac{\alpha (x,t) \Delta {t}}{2h^2}\times \nonumber \\ & \sum \limits ^{{\mathscr {N}+4}}_{{j=\mathscr {N}-4}}v^{k}_{j}a^{H}_{\mathscr {N}-j}\mathscr {D}^{''}(\mathscr {N}-j)+ \Delta t \xi ^k_{\mathscr {N}}(x_\mathscr {N},t), \end{aligned}$$

By combining the equations (17–20) we get

21 $$\begin{aligned} U^{k+1}-\frac{\alpha (x,t) \Delta {t}}{2h^2}\mathscr {H}_{1}\mathscr {V}^{k+1}=U^{k}+\frac{\alpha (x,t) \Delta {t}}{2h^2}\mathscr {H}_{2}\mathscr {V}^{k}+F^{k}, \end{aligned}$$

by using equation (8) to make some simplifications, equation (21) can be modified as

22 $$\begin{aligned} -\mathscr {G}_{1}\mathscr {V}^{k+1}=\mathscr {G}_{2}\mathscr {V}^{k}+F^k, \end{aligned}$$

this implies

23 $$\begin{aligned} \mathscr {V}^{k+1}=\mathscr {E}\mathscr {V}^{k}+{\mathscr {F}^{k}},\qquad where \qquad \mathscr {E}={-\mathscr {G}_{1}^{-1}}\mathscr {G}_{2}, \qquad {\mathscr {F}}={\mathscr {G}_{1}^{-1}}F^k. \end{aligned}$$

Where

$$\begin{aligned} \begin{array}{l} \mathscr {V}^{k}=(v^{k}_{-4},v^{k}_{-3},\cdots , v^{k}_{\mathscr {N}+3}, v^{k}_{\mathscr {N}+4})^{T}, \,\ \& \,\ \mathscr {V}^{k+1}=(v^{k+1}_{-4},v^{k+1}_{-3}, \cdots , v^{k+1}_{\mathscr {N}+3},,v^{k+1}_{\mathscr {N}+4})^{T}\\ \\ \mathscr {F}= \Delta t (0,0,0,g_{1}(t),f^k_{0}(x_0,t),\cdots ,f^k_{\mathscr {N}}(x_\mathscr {N},t),g_{2}(t),0,0,0)^{T} \end{array} \end{aligned}$$

for $1 \le e\le \mathscr {N}+1$ and $1\le b\le \mathscr {N}+9$, matrix $\mathscr {G}_{1}$ of order ${(\mathscr {N}+1)\times (\mathscr {N}+9)}$ is defined below

24 $$\begin{aligned} \mathscr {G}_{1}=[\Psi ^{(eb)}_{b-e-4}] \end{aligned}$$

with

$$\begin{aligned} \Psi (l)=\left\{ \begin{array}{l} \mathscr {D}''_{l}, \quad \text{ if }\quad -4\le l<0 \quad \text{ and }\quad 0<l \le 4\\ 0, \qquad \text{ if }\quad l\notin [-4,4]\\ a^{H}_0\mathscr {D}''_{l}-\frac{2a^{H}_0 h^2}{{\alpha (x, t)} \Delta t}, \qquad \text{ if }\quad l=0 \end{array}\right. \end{aligned}$$

similarly $\mathscr {G}_{2}$ of order ${(\mathscr {N}+1) \times (\mathscr {N}+9)}$ is given below

25 $$\begin{aligned} \mathscr {G}_{2}=[\Omega ^{(eb)}_{b-e-4}] \end{aligned}$$

with

$$\begin{aligned} \Omega (l)=\left\{ \begin{array}{l} \mathscr {D}''_{l}, \quad \text{ if }\quad -4\le l<0 \quad \text{ and }\quad 0<l \le 4\\ 0, \qquad \text{ if }\quad l\notin [-4,4]\\ a^{H}_0\mathscr {D}''_{l}+\frac{2a^{H}_0 h^2}{{\alpha (x, t)} \Delta t}, \qquad \text{ if }\quad l=0 \end{array}\right. \end{aligned}$$

### Forced conditions

We need eight additional conditions in order to have a unique solution of (23). So, at the left end of the domain, we’ll build four conditions, and at the right end of the domain, we’ll build four conditions.

The left and right end points are represented by $v_ {-3},v_ {-2}, v_ {-1}$ and $v_ {\mathscr {N}+1}, v_ {\mathscr {N}+2}, v_ {\mathscr {N}+3}$, respectively. For $0\le \tilde{m}\le 3$, these initial points and final points are calculated using a polynomial of degree four that interpolates the data $(u_{\tilde{m}}, c_{\tilde{m}})$. i.e.

The left end conditions are obtained from

$$\begin{aligned} v_{-\tilde{m}}=B(-u_{\tilde{m}}), \qquad \tilde{m}=1,2,3, \end{aligned}$$

where

26 $$\begin{aligned} B(u_{\tilde{m}})=\sum ^{6}_{\tilde{r}=1}\left( {\begin{array}{c}6\\ \tilde{r}\end{array}}\right) (-1)^{\tilde{r}+1}\mathbb {V}(u_{\tilde{m}-\tilde{r}}). \end{aligned}$$

Since by (8), $\mathbb {V}(u_{\tilde{m}})=v_{\tilde{m}}$ for $\tilde{m}=1,2,3$ and replacing $u_{\tilde{m}}$ by $-u_{\tilde{m}}$ in (26), we have

27 $$\begin{aligned} \sum ^{6}_{\tilde{r}=0}\left( {\begin{array}{c}6\\ \tilde{r}\end{array}}\right) (-1)^{\tilde{r}}v_{\tilde{r}-\tilde{m}}=0, \tilde{m}=3,2,1. \end{aligned}$$

Similarly, we have following three right end conditions

28 $$\begin{aligned} \sum ^{6}_{\tilde{r}=0}\left( {\begin{array}{c}6\\ \tilde{r}\end{array}}\right) (-1)^{\tilde{r}}v_{\tilde{m}-\tilde{r}}=0,\,\ \tilde{m}=\mathscr {N}+3,\mathscr {N}+2,\mathscr {N}+1. \end{aligned}$$

After employing these three left and right end conditions we have the following consistent system

29 $$\begin{aligned} \mathscr {V}^{k+1}=\mathscr {J}_{v}\mathscr {V}^{k}+\mathscr {F}^{k} \end{aligned}$$

where

30 $$\begin{aligned} \mathscr {J}_{v}=(\mathscr {L}_{v_{0}}^T, \mathscr {E}^T, \mathscr {R}_{v_{\mathscr {N}}}^T){^T}. \end{aligned}$$

In the resulting matrix $\mathscr {J}_{v}$, four left and right boundary conditions are represented by $\mathscr {L}_{ v_ {0}}$ and $\mathscr {R}_{v_{\mathscr {N}}}$ respectively. Starting three rows of $\mathscr {L}_{ v_ {0}}$ are derived from (27). While the last row of $\mathscr {L}_{v_{0}}$ is formed from (3) at $\mathscr {V}(0)=v_{0}=g_{1}(t)$. Similarly the last three rows of the matrix $\mathscr {R}_{v_{\mathscr {N}}}$ are derived from (28) and first row comes from (3) at $\mathscr {V}(1)=v_{\mathscr {N}}=g_{2}(t)$. Hence the column vector $\mathbb {M}$ is defined as

31 $$\begin{aligned} \mathbb {M}=(0, 0, 0, \mathscr {V}(0), {(\mathscr {V}^{k+1})}^{T}, \mathscr {V}(\mathscr {N}), 0, 0, 0)^{T} \end{aligned}$$

where $\mathscr {V}^{k+1}$ is given in (23).

### Initial solution

Iterative procedure is required to solve the problem to start iteration we need initial vector

$V^0=[v_{-4}, v_{-3}, v_{-2},v_{-1},...,v_{\mathscr {N}+1},v_{\mathscr {N}+2},v_{\mathscr {N}+3},v_{\mathscr {N}+4}]^T$, this initial vector is obtained by initial and boundary conditions discussed as follows

32 $$\begin{aligned} u(x,0)=\sum \limits ^{\mathscr {N}+4}_{p=-4}v_{p}\mathscr {S}_{2,p} \qquad 0\le x\le \mathbb {L}, \end{aligned}$$

where ${v_{p}}'s$ are unknown. Following is the required conditions which must satisfy the initial approximation *u*(*x*, 0)

$$\begin{aligned} u(x_p,0)= & {h}(x_p), p=0,1...,\mathscr {N} \\ u(x_0,0)= & g_{1}(t)=0,\qquad u(x_\mathscr {N},0)=g_{2}(t)=0 \end{aligned}$$

Consequently we get a matrix system of order $(\mathscr {N}+9)\times (\mathscr {N}+9)$

33 $$\begin{aligned} \mathscr {J}_{v} \mathscr {V}^{0}+\mathscr {F}^{0}=\mathscr {H} \end{aligned}$$

is obtained where $\mathscr {J}_{v}$ is defined in (30) and $\mathbb {H}=[0,0,0,0,g_{1}(x_0),...,g_{2}(x_\mathscr {N}),0,0,0,0]^T$ is a column matrix of $(\mathscr {N}+9)$ rows.

## Stability analysis and errors evaluation

This part demonstrate the stability and errors evaluation of the proposed algorithm.

### Stability analysis

#### Theorem 1

The iterative algorithm (29) obtained using subdivision collocation algorithm converges for $h \ge 0$ and $\Delta t\ge 0$ where $\eta \in [-\pi ,\pi ]$.

#### Proof

The von Neuman stability analysis uses Fourier series to decompose numerical approximation errors. The Fourier series decomposes any periodic function or signal into the sum of a set of simple oscillating functions, mainly sines and cosines. Complex exponentials are far more convenient to represent errors than real trigonometric functions. So we have,

34 $$\begin{aligned} \mathscr {V}^{\tilde{n}}_{\lambda }=\exp (\imath \eta \lambda h), \end{aligned}$$

We consider

35 $$\begin{aligned} \sum \limits ^{4}_{\lambda =-4}\mathscr {D}''({\lambda })\mathscr {V}^{k+1}_{\lambda }= \sum \limits ^{4}_{\lambda =-4}\mathscr {D}''({\lambda })^{*}\mathscr {V}^{k}_{\lambda }. \end{aligned}$$

Substitution of (34) in (35) implies

$$\begin{aligned} \sum \limits ^{4}_{\lambda =-4}\mathscr {D}''({\lambda })\exp (i\eta \lambda h)=\sum \limits ^{4}_{\lambda =-4}\mathscr {D}''({\lambda })^{*}\exp (i\eta \lambda h) \end{aligned}$$

after simplification we get the result

36 $$\begin{aligned} |\xi |=\left| \frac{\mathbb {A}+i\mathbb {B}}{\mathbb {A}'+i\mathbb {B}'}\right| \le 1, \end{aligned}$$

where

$$\begin{aligned} & \mathbb {A}=\frac{3}{280}cos4\eta h+\frac{8}{35}cos3\eta h-\frac{184}{105}cos2\eta h+\frac{712}{105}cos\eta h-\frac{295}{28}+\frac{2a_{0}{^{H}}h^{2}}{(\alpha (x,t) \Delta t)^2},\\ & \mathbb {B}=0, \end{aligned}$$

and

$$\begin{aligned} & \mathbb {A}'=\frac{3}{280}cos4\eta h+\frac{8}{35}cos3\eta h-\frac{184}{105}cos2\eta h+\frac{712}{105}cos\eta h-\frac{295}{28}+\frac{2a_{0}{^{H}}h^{2}}{(\alpha (x,t) \Delta t)^2}, \\ & \mathbb {B}'=0. \end{aligned}$$

Using values of $\mathbb {A}, \mathbb {A}' , \mathbb {B} \quad and \quad \mathbb {B}'$ in equation (36), simplification ensures $|\xi |\le 1$ for $0<a^{H}_{0}<1$,$h\ge 0$ and $\Delta t\ge 0$, $\eta \in [-\pi ,\pi ]$. $\square$

### Errors evaluation

Let $\mathscr {U}(x)$ be the exact solution and *u*(*x*) be the numerical solution of the (1). Then different errors between exact solutions and approximate solutions are estimated as

$$\begin{aligned} AE= & \parallel \mathscr {U}_{i}-u_{i}\parallel .\\ L^{\infty }= & \parallel \mathscr {U}_{i}-u_{i}\parallel _{\infty }. \\ L^{2}= & \parallel \mathscr {U}_{i}-u_{i}\parallel _{2}. \end{aligned}$$

Similarly average errors ($E_{AVG}$) and root mean square errors ($E_{RMS}$) are estimated as

$$\begin{aligned} E_{AVG}= & \frac{\sum \limits _{i=1}^{\mathscr {N}}\parallel \mathscr {U}_{i}-u_{i}\parallel _{\infty }}{\mathscr {N}}. \\ E_{RMS}= & \sqrt{\frac{\sum \limits _{i=1}^{\mathscr {N}}\parallel \mathscr {U}_{i}-u_{i}\parallel _{\infty }}{\mathscr {N}}}. \end{aligned}$$

The maximum relative error $(E_{MR})$ is estimated as

$$\begin{aligned} E_{MR}=\parallel \frac{\mathscr {U}_{i}-u_{i}}{\mathscr {U}_{i}}\parallel _{\infty }. \end{aligned}$$

## Comparison of numerical example with existing methods

In this part we find solution of the one-dimensional heat equation (1) for different values of $\alpha (x,t)$, that guarantees the existence of unique solutions using subdivision collocation algorithm. We’ve also made tables of comparison for the various values of $\alpha (x,t)$ to demonstrate the accuracy of the proposed technique in comparison to the analytical solution and other approaches solutions. Matlab was used to carry out the computations.

### Numerical examples

#### Example 1

We consider (1) taken from^1^, with ${{\alpha }(x,t)}=\frac{x}{2}(1-x),\mathbb {L}=1, {{h}}(x)=x(1-x)$, heat source $\xi (x,t)=0$. ${g}_{1}(t), {g}_{2}(t)$ can be obtained from the exact solution which is $x(1-x)e^{-t}$.

#### Example 2

We consider (1) taken from^1^, with ${{\alpha }(x,t)}=\frac{tx}{2}(1-x),\mathbb {L}=1$, h$(x)=x(1-x)$, heat source $\xi (x,t)=0$. ${g}_{1}(t), {g}_{2}(t)$ can be obtained from the exact solution which is $x(1-x)e^{-t^2}$.

#### Example 3

We consider (1) taken from^1^, with ${{\alpha }(x,t)}=\frac{x^2}{2},\mathbb {L}=1,{{h}}(x)=x^2$, heat source $\xi (x,t)=0$. ${g}_{1}(t), {g}_{2}(t)$ can be obtained from the exact solution which is $x^2 exp(t)$.

#### Example 4

We consider (1) taken from^1^, with ${{\alpha }(x,t)}=x^2 t,\mathbb {L}=1, {{h}}(x)=x^2$, heat source $\xi (x,t)=0$. ${g}_{1}(t), {g}_{2}(t)$ can be obtained from the exact solution which is $x^2 exp(t^2)$.

## Results and discussions

Numerical results of Examples 1−4 are being presented in this section.

**Fig. 1:**
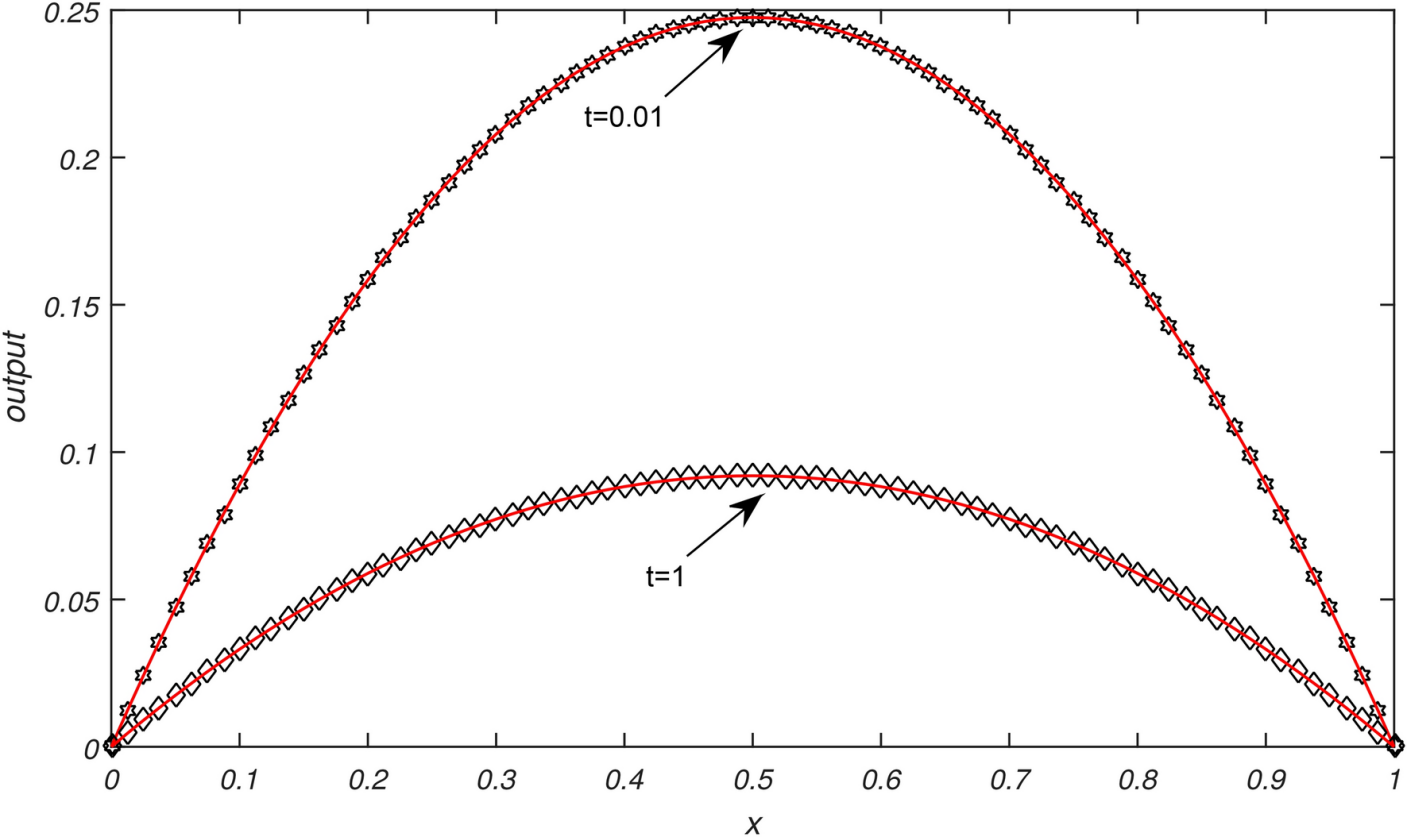
Graphical illustration of exact and approximate solutions of Example 1 for $N=79$, $\Delta t=0.0125$ at different values of *t*.

**Fig. 2:**
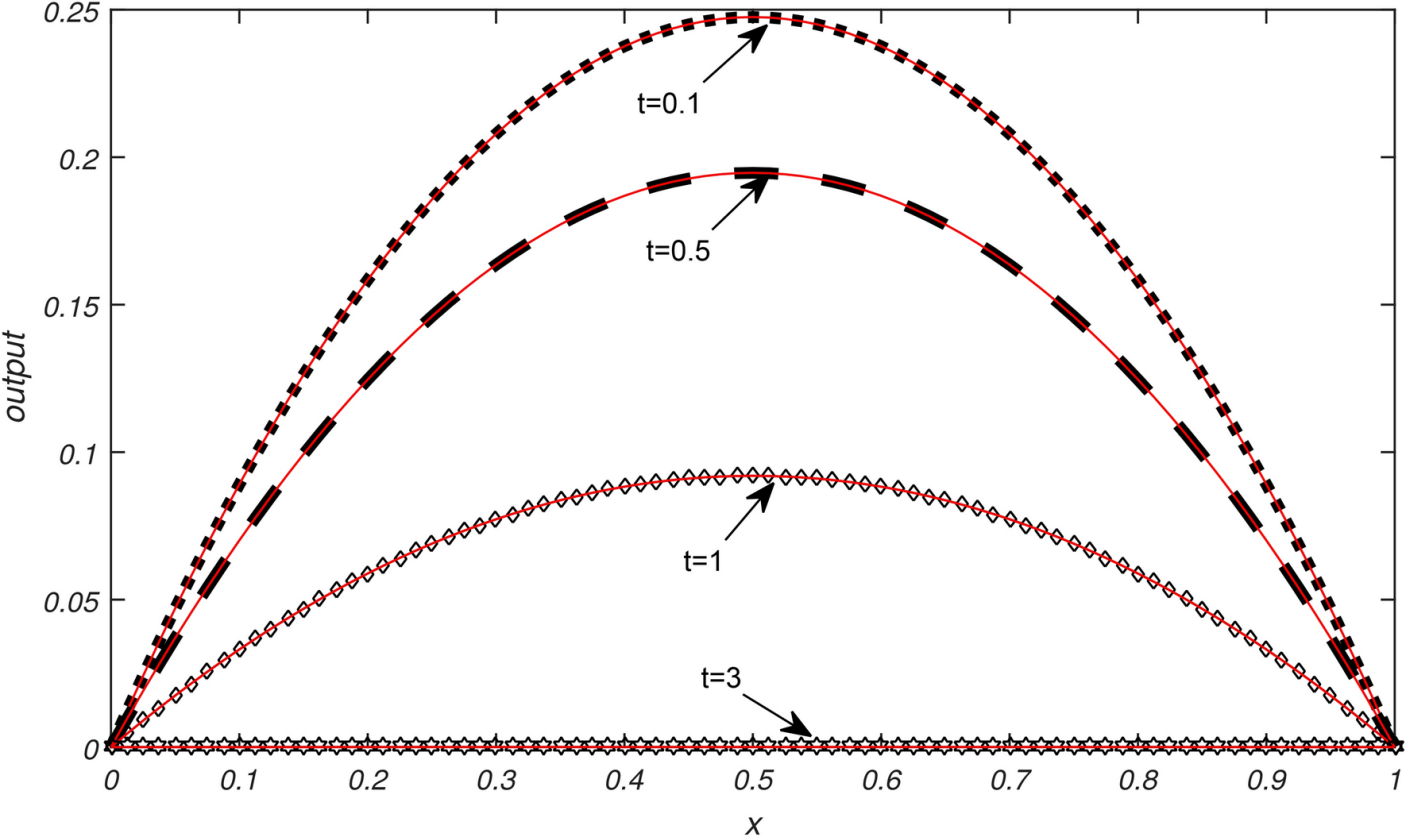
Graphical illustration of exact and approximate solutions of Example 2 for $N=79, \Delta t=0.0125$ at different values of *t*..

**Fig. 3:**
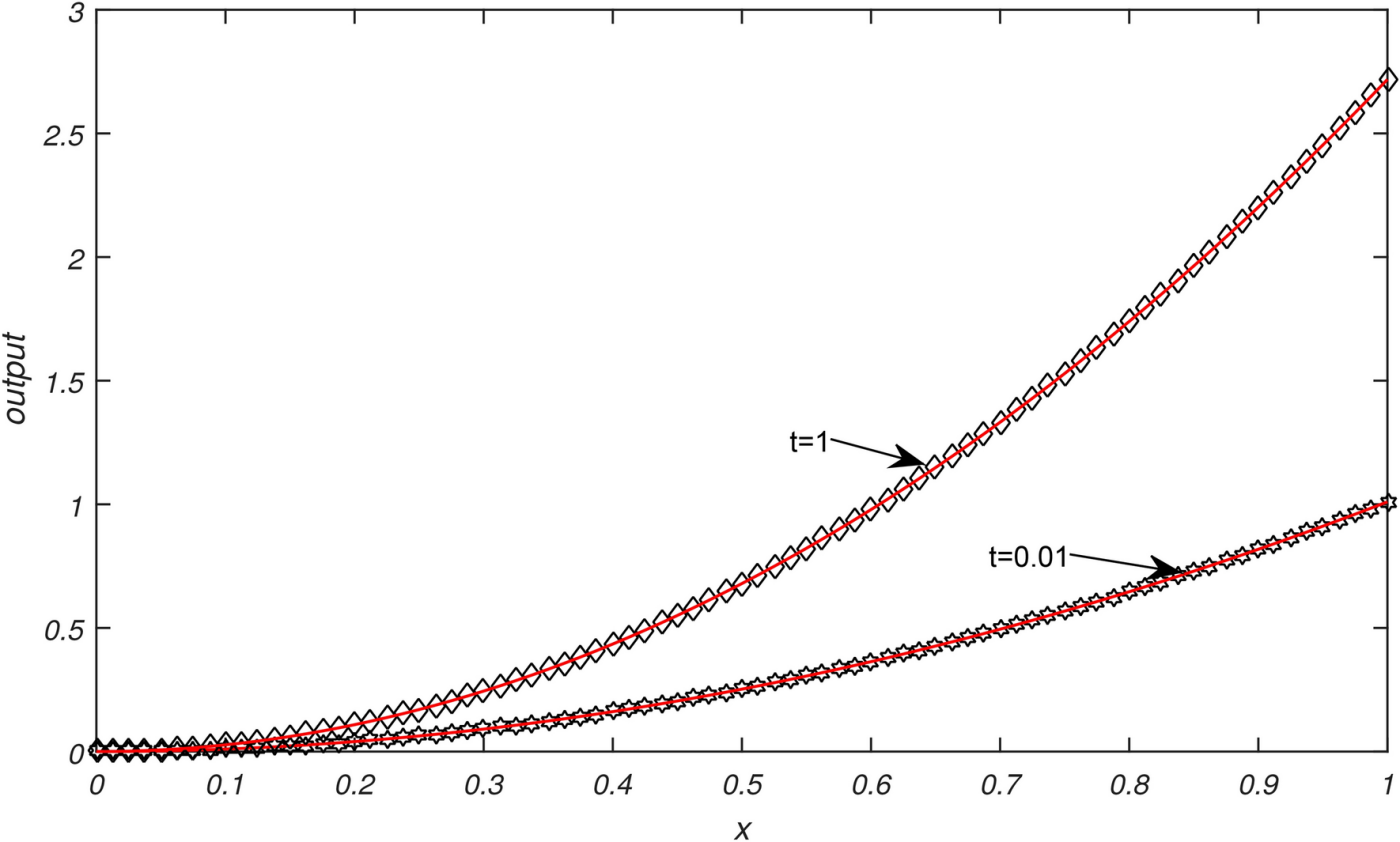
Graphical illustration of exact and approximate solutions of Example 3 for $N=79, \Delta t=0.0125$ at different values of *t*.

**Fig. 4:**
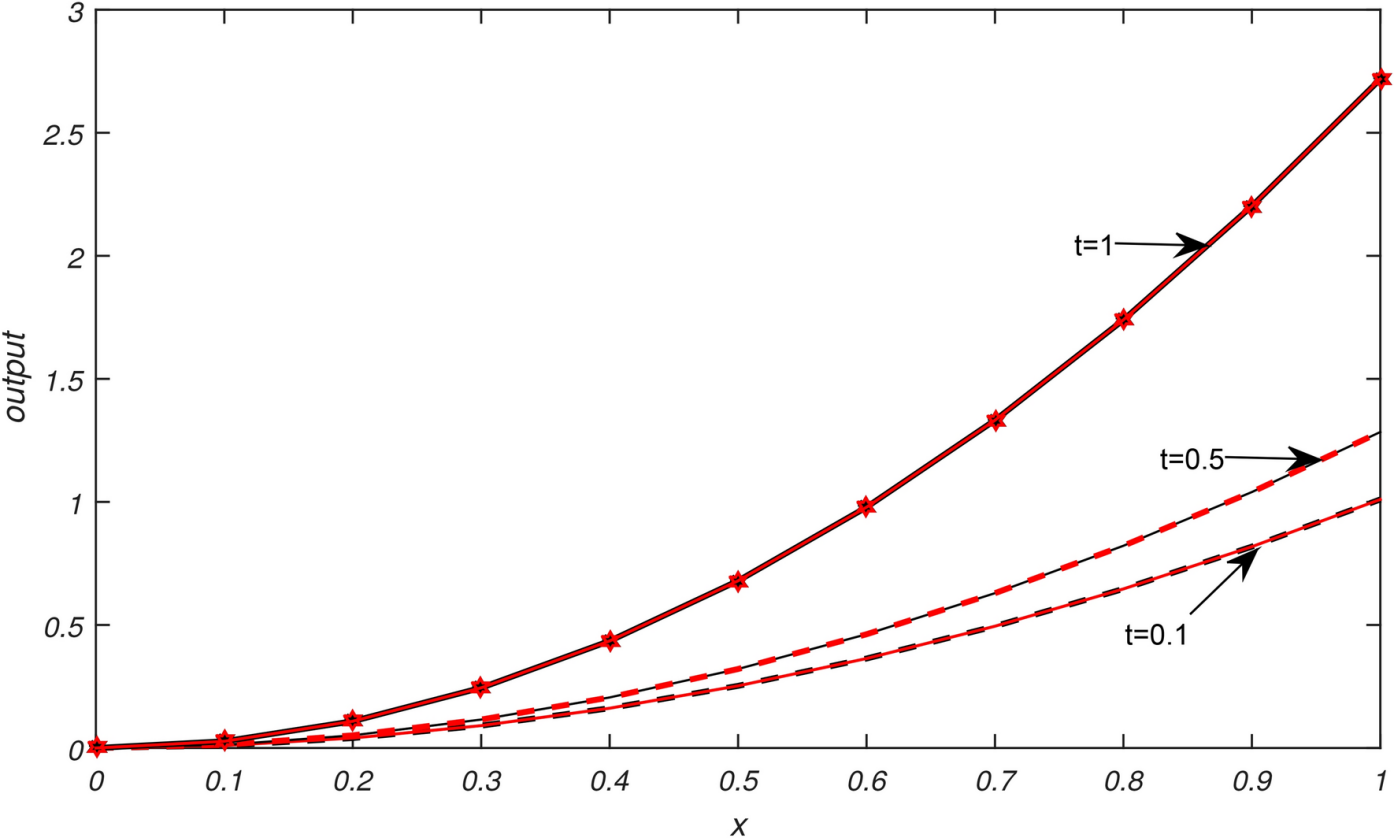
Graphical illustration of exact and approximate solutions of Example 4 for $N=79, \Delta t=0.0125$ at different values of t.

- Example 1 is solved with non uniform thermal diffusivity which varies along spatial coordinate. Numerical data for $N=9,19,39,79$, $\Delta t= 0.1, 0.05, 0.025,0.0125$ and $h= 0.1, 0.05, 0.025,0.0125$ is presented in Table 1. These obtained results using SCA are compared with the existing numerical methods of^1^. Graphical representation of Example 1 is given in Fig. 1 for $N=79, \Delta t=0.0125, t=1\,\ \& \,\ t=0.01$. In Fig. 1, exact solution is represented by black color and approximate solution by red color. From Fig. 1 it is observed that temperature distribution varied quadratically along space coordinate, decay exponentially over the time. It is clear from graphical and numerical results that the proposed method presented in this research work is more efficient than the methods of^1^.
- Example 2 is solved with non uniform thermal diffusivity which varies along spatial coordinate when temporal variations occur. Numerical data for $N=9,19,39,79$, $\Delta t= 0.1, 0.05, 0.025, 0.0125$, $h= 0.1, 0.05, 0.025, 0.0125$ is presented in Table 2. These obtained results using SCA are compared with the existing numerical methods of^1^. Graphical representation of Example 2 with temporal variation is given in Fig. 2 for $N=79$, $\Delta t=0.0125$ and $t=1 \,\ \& \,\ t=0.01$. In Fig. 2, exact solution is represented by black color and approximate solution by red color. Also graphical behaviour shows that the temperature distribution varies quadratically, it increases rapidly with the position from origin and decay exponentially over time. It is clear from graphical and numerical results that the current method converges rapidly and gives more smooth results and higher approximation order than the methods of^1^.
- Example 3 is solved with non uniform thermal diffusivity which varies along spatial coordinate. Numerical data for $N=9,19,39,79$, $\Delta t= 0.1, 0.005, 0.025,0.0125$ and $h= 0.1, 0.005, 0.025,0.0125$ is presented in Table 3. These obtained results using SCA are compared with the existing numerical methods of^1^. Graphical representation of Example 3 is given in Fig. 3 for $N=79, \Delta t=0.0125, t=1 \,\ \& \,\ t=0.01$. In Fig. 3, exact solution is represented by black color and approximate solution by red color. Here temperature distribution is parabolic and symmetric along position, increases exponentially as time progresses. It is evident from the graphical and numerical results that approximation order of current method is higher that the existing methods of^1^. The proposed method provides efficient and smooth results than the methods of^1^.
- Example 4 is solved with non uniform thermal diffusivity which varies along spatial coordinate when temporal variations occur. Numerical data for $h=0.1$, $\Delta t= 0.001,0.002,0.003,0.004,0.005$ and $t=0.5$ is presented in Table 4. These obtained results using SCA are compared with the existing numerical methods of^1^. Graphical representation of Example 4 with temporal variation is given in Fig. 4 for $N=79$, $\Delta t=0.0125$. In Fig. 4, exact solution is represented by black color and approximate solution by red color. Also graphical behaviour shows that temperature distribution increases in space and with time it grows exponentially. It is obvious from the graphical and numerical results that the current method is more efficient than the methods of^1^.
- Table 5 represents the different errors which shows the accuracy of the proposed algorithm.

**Table 1:** Maximum absolute errors of Example 1.

	by SCA				by^1^			
$\Delta t$	$N=9$	$N=19$	$N=39$	$N=79$	$N=9$	$N=19$	$N=39$	$N=79$
0.1	2.36 × 10^−16^	7.49 × 10^−16^	8.33 × 10^−17^	9.71 × 10^−17^	0.23 × 10^−5^	0.23 × 10^−5^	0.23 × 10^−5^	0.23 × 10^−5^
0.05	5.13 × 10^−16^	2.33 × 10^−15^	5.55 × 10^−17^	8.33 × 10^−17^	0.29 × 10^−6^	0.29 × 10^−6^	0.29 × 10^−6^	0.29 × 10^−6^
0.025	1.10 × 10^−15^	1.26 × 10^−15^	9.71 × 10^−17^	1.67 × 10^−16^	0.37 × 10^−7^	0.37 × 10^−7^	0.37 × 10^−7^	0.37 × 10^−7^
0.0125	2.40 × 10^−15^	3.00 × 10^−15^	1.04 × 10^−16^	5.55 × 10^−17^	0.48 × 10^−8^	0.48 × 10^−8^	0.48 × 10^−8^	0.48 × 10^−8^

**Table 2:** Maximum absolute errors of Example 2.

	by SCA				by^1^			
$\Delta t$	$N=9$	$N=19$	$N=39$	$N=79$	$N=9$	$N=19$	$N=39$	$N=79$
0.1	2.36 × 10^−16^	7.49 × 10^−16^	8.33 × 10^−17^	9.71 × 10^−17^	0.69 × 10^−5^	0.69 × 10^−5^	0.69 × 10^−5^	0.69 × 10^−5^
0.05	5.13 × 10^−16^	2.33 × 10^−15^	5.55 × 10^−17^	8.33 × 10^−17^	0.91 × 10^−6^	0.91 × 10^−6^	0.91 × 10^−6^	0.91 × 10^−6^
0.025	1.10 × 10^−15^	1.26 × 10^−15^	9.71 × 10^−17^	1.67 × 10^−16^	0.12 × 10^−6^	0.12 × 10^−6^	0.12 × 10^−6^	0.12 × 10^−6^
0.0125	2.40 × 10^−15^	3.00 × 10^−15^	1.04 × 10^−16^	5.55 × 10^−17^	0.15 × 10^−7^	0.15 × 10^−7^	0.15 × 10^−7^	0.15 × 10^−7^

**Table 3:** Maximum absolute errors of Example 3.

	by SCA				by^1^			
$\Delta t$	$N=9$	$N=19$	$N=39$	$N=79$	$N=9$	$N=19$	$N=39$	$N=79$
0.1	3.11 × 10^−15^	3.13 × 10^−14^	5.77 × 10^−15^	7.11 × 10^−15^	0.26 × 10^−4^	0.26 × 10^−4^	0.26 × 10^−4^	0.26 × 10^−4^
0.05	4.22 × 10^−15^	1.03 × 10^−13^	3.55 × 10^−15^	5.33 × 10^−15^	0.39 × 10^−5^	0.40 × 10^−5^	0.40 × 10^−5^	0.40 × 10^−5^
0.025	2.04 × 10^−14^	7.95 × 10^−14^	4.44 × 10^−15^	1.29 × 10^−14^	0.57 × 10^−6^	0.59 × 10^−6^	0.59 × 10^−6^	0.59 × 10^−6^
0.0125	4.13 × 10^−14^	1.26 × 10^−13^	1.15 × 10^−14^	8.88 × 10^−15^	0.83 × 10^−7^	0.85 × 10^−7^	0.85 × 10^−7^	0.84 × 10^−7^

**Table 4:** Maximum absolute errors of Example 4, at t=0.5.

$\Delta t$	h=0.1, by SCA	h=0.1, by^1^
0.005	1.18 × 10^−13^	0.68 × 10^−3^
0.004	1.81 × 10^−13^	0.35 × 10^−3^
0.003	1.96 × 10^−13^	0.21 × 10^−2^
0.002	2.94 × 10^−13^	0.55 × 10^−4^
0.001	5.88 × 10^−13^	0.74 × 10^−5^

**Table 5:** Different errors of Examples 1–4, by SCA for $\Delta t=h, N=50, t=1$.

Example	$L^{\infty }$	$L^{2}$	$E_{AVG}$	$E_{RMS}$	$E_{MR}$
1	2.22 × 10^−15^	3.37 × 10^−15^	3.00 × 10^−16^	4.77 × 10^−16^	8.17 × 10^−16^
2	2.22 × 10^−15^	3.25 × 10^−15^	2.51 × 10^−16^	4.59 × 10^−16^	8.17 × 10^−16^
3	8.33 × 10^−17^	2.16 × 10^−16^	2.36 × 10^−17^	3.05 × 10^−17^	9.05 × 10^−16^
4	8.33 × 10^−17^	2.16 × 10^−16^	2.36 × 10^−17^	3.05 × 10^−17^	9.05 × 10^−16^

## Conclusion

In this research, we developed a collocation approach for solving the one-dimensional heat equation with non uniform thermal diffusivity using a subdivision scheme. The numerical results revealed that utilising the subdivision collocation technique for the approximate solution of heat conduction equation (1), the approach is appropriate. We concluded that the numerical results of the proposed problem converge to the precise solution for the tiny step size based on these findings. We also compared the absolute errors of the solution generated by the subdivision collocation algorithm with those produced by the numerical methods presented in^1^. In comparison to other current approaches, we find that our algorithm produces less absolute errors than those of^1^.

## Data Availability

The data used to support the findings of the study are available within this paper.”
